# Development and validation of the 9-item Patient Satisfaction Questionnaire (PSQ-9) for use in community pharmacy settings

**DOI:** 10.1080/20523211.2026.2686185

**Published:** 2026-06-17

**Authors:** Fouad Sakr, Mariam Dabbous, Jihan Safwan, Mona El Bakri, Mohamad Rahal, Pascale Salameh

**Affiliations:** a School of Pharmacy, Lebanese International University, Beirut, Lebanon; b INSPECT-LB: Institut National de Santé Publique, d'Épidémiologie Clinique et de Toxicologie-Liban, Beirut, Lebanon; c Inserm U1094, IRD UMR270, Univ. Limoges, CHU Limoges, EpiMaCT – Epidemiology of chronic diseases in tropical zone, Institute of Epidemiology and Global Health – Michel Dumas, OmegaHealth, Limoges, France; d Faculty of Pharmacy, Lebanese University, Beirut, Lebanon; e Gilbert and Rose-Marie Chagoury School of Medicine, Lebanese American University, Byblos, Lebanon; f Department of Primary Care and Population Health, University of Nicosia Medical School, Nicosia, Cyprus

**Keywords:** Patient satisfaction, community pharmacy, PSQ-9, validation

## Abstract

**Background:**

Patient satisfaction is a key indicator of healthcare quality and a driver of medication adherence and trust in community pharmacy practice. However, validated instruments that specifically assess patients’ satisfaction with community pharmacists and pharmacies remain scarce. This study aimed to develop and validate the 9-item Patient Satisfaction Questionnaire (PSQ-9), a concise tool tailored to community pharmacy settings, and to identify factors associated with patient satisfaction.

**Methods:**

A cross-sectional online survey of 501 adults from all Lebanese districts was conducted between February and May 2025. The PSQ-9 was derived from the modified 18-item PSQ using exploratory (EFA) and confirmatory (CFA) factor analyses on two independent subsamples. Psychometric testing included internal consistency, test-retest reliability, construct validity, and multigroup CFA to assess measurement invariance. Multivariable linear regression identified correlates of satisfaction.

**Results:**

The PSQ-9 demonstrated a two-factor structure, Interpersonal Care and Professional Engagement and Affordability and Informational Assurance, with excellent model fit (CFI = 0.984, RMSEA = 0.056, SRMR = 0.027) and reliability (α = 0.904, ω = 0.910, ICC = 0.729). Measurement invariance was established across gender, health status, and access to care. Higher satisfaction was associated with easier access to healthcare (B = 1.470, *P* = 0.012), pharmacist counselling from time to time (B = 1.616, *P* = 0.004) or regularly (B = 1.864, *P* = 0.008), longer counselling (>10 min; B = 2.441, *P* = 0.004), higher patient expectations (B = 0.356, *P* = 0.001) and perceptions (B = 0.110, *P* = 0.002), and fewer communication barriers (B = –0.235, *P* = 0.005).

**Conclusion:**

The PSQ-9 is a valid, reliable, and contextually adapted instrument for assessing patient satisfaction with community pharmacy services. Its brevity and psychometric robustness make it ideal for large-scale surveys and benchmarking, supporting patient-centered and equitable pharmacy practice improvement.

## Background

Patient satisfaction is a key indicator of healthcare quality, reflecting patients’ perceptions of service delivery, provider-patient interaction, and the gap between expectations and experience (Ferreira et al., 2023). In community pharmacy settings, satisfaction plays a critical role in fostering trust, improving medication adherence, and encouraging continued use of pharmaceutical services (Molla et al., 2022; Traverso & MacKeigan, 2005). As pharmacists expand their role from traditional dispensing toward patient-centered care and medication management, the need for valid and reliable measures of satisfaction becomes increasingly important (McFarland et al., 2021; Moon et al., 2016).

Despite this importance, psychometrically validated tools assessing patients’ satisfaction specifically with community pharmacists and pharmacies remain scarce (Resende et al., 2022). Most available instruments were developed for medical or hospital contexts and may not adequately capture dimensions unique to the pharmacist-patient relationship (Carpenter et al., 2021). Tools with lengthy item sets or multiple domains increase respondent burden and reduce feasibility for routine or large-scale surveys (Amirthalingam et al., 2024). Moreover, in low- and middle-income countries, contextual factors such as affordability, accessibility, and health coverage variability necessitate culturally adapted and psychometrically robust measures (Iskandar et al., 2017; Molla et al., 2022).

Several pharmacy-specific satisfaction instruments have been proposed. The Pharmaceutical Care Satisfaction Questionnaire (PCSQ), for instance, includes 30 items assessing multiple dimensions of pharmaceutical care services (Gourley et al., 2001). Moreover, a recently developed 24-item instrument demonstrated a four-factor structure for assessing satisfaction with pharmaceutical services in ambulatory care (Huang et al., 2023). Despite their usefulness, these tools often suffer from excessive length, limited cultural adaptability, and incomplete psychometric testing, making them impractical for rapid or routine evaluation in community settings. Moreover, existing literature highlight substantial heterogeneity and a lack of standardisation among pharmacy satisfaction scales (Sakharkar et al., 2015; Traverso & MacKeigan, 2005).

In Lebanon, community pharmacies operate within a fragmented health system characterised by economic instability, limited insurance coverage, and fluctuating medication availability, all of which influence patient experiences (Iskandar et al., 2017). The IMPHACT-LB study found that approximately 60% of patients were satisfied with pharmacy services and identified socioeconomic disparities as major determinants of satisfaction (Sacre et al., 2023). Similar studies have shown variations by education, income, and regional access, underscoring the impact of systemic and contextual factors on satisfaction levels (Ismail et al., 2020).

Given these limitations, particularly the need for brevity, cultural relevance, and rigorous validation, a concise, psychometrically sound instrument to assess community pharmacy satisfaction is warranted. A short scale would minimise respondent burden, facilitate repeated use in surveys, and enhance applicability in both research and quality monitoring. This approach aligns with best practices in scale development, including item reduction, exploratory and confirmatory factor analyses, and measurement invariance testing (Boateng et al., 2018; Larson et al., 2002). Therefore, the present study aimed to develop and validate the 9-item Patient Satisfaction Questionnaire (PSQ-9) tailored for community pharmacy settings, and to examine its validity, reliability, and correlates of patient satisfaction across sociodemographic, economic, clinical, and patient-reported experience dimensions.

## Methods

### Study design and participants

A cross-sectional study was conducted in Lebanon between February and May 2025 to assess patient satisfaction with community pharmacy services. Adults aged 18 years or older who had obtained care from a community pharmacist or pharmacy in the past six months were recruited through snowball sampling. The questionnaire was distributed online via Google Forms and shared across social media platforms (WhatsApp, Facebook, Instagram, and LinkedIn) to ensure representation from all Lebanese districts.

The questionnaire introduction described the study objectives, approximate completion time, and voluntary nature of participation, highlighting the option to withdraw at any point. Participants who consented to provide an email or phone number for follow-up were recontacted in May 2025 to complete the retest phase for reliability assessment; those who declined were excluded.

A pilot test with 10 individuals confirmed the clarity and comprehension of the survey, leading to minor revisions before full deployment. Data from the pilot were excluded from analyses. Contact details were used solely to match test-retest responses and were removed from the analytical dataset to maintain confidentiality.

### Measures and variables

The study questionnaire was in Arabic and comprised five sections. The first collected sociodemographic and socioeconomic data, including age, gender, area of residence, education level, employment status, and monthly income. Financial well-being was evaluated using the 8-item InCharge Financial Distress/Financial Well-Being (IFDFW) scale, rated from 1 (lowest) to 10 (highest), where higher scores denote better financial well-being (Prawitz et al., 2006). In this sample, Cronbach’s α = 0.960.

The second section addressed clinical characteristics, such as current health status, presence and type of chronic conditions, medication use, access to healthcare, and type of health coverage. Medication adherence was assessed using the Lebanese Medication Adherence Scale (LMAS-14), a validated 14-item tool scored on a 4-point Likert scale (1 = low adherence to 4 = high adherence); higher total scores reflect better adherence (Bou Serhal et al., 2018; Sakr et al., 2022) (α = 0.934).

The third section explored patient experiences in community pharmacies using the Patient-Pharmacist Relationship Measurement Tool, validated in Lebanon (Sacre et al., 2024). It includes three indices: the Patient Expectation Index (PEI) (11 items; α = 0.745), the Barriers to Communication Index (BCI) (7 items; α = 0.905), and the Patient Perception Index (PPI) (14 items; α = 0.905). Higher scores represent greater expectations, perceived barriers, or positive perceptions, respectively.

The fourth section measured patient satisfaction through the validated modified 18-item Patient Satisfaction Questionnaire Short Form (MA-PSQ18) (Sacre et al., 2023). The instrument evaluates satisfaction with community pharmacies and pharmacists across domains such as general satisfaction, technical quality, interpersonal manner, time spent with the pharmacist, and accessibility/convenience. Subscale and total scores are calculated by summing item responses, with higher values indicating greater satisfaction (α = 0.952).

The fifth section assessed health-related quality of life using the EQ-5D-5L, which includes five dimensions (mobility, self-care, usual activities, pain/discomfort, and anxiety/depression) and the EQ Visual Analogue Scale (EQ VAS) ranging from 0 (worst) to 100 (best imaginable health) (Xu et al., 2024).

For the test-retest phase, the MA-PSQ18 and LMAS-14 were re-administered. The MA-PSQ18 retest was part of this study, while the LMAS-14 retest contributed to a related validation project within the same research framework.

### Development and content validity of the 9-item Patient Satisfaction Questionnaire (PSQ-9)

The 9-item Patient Satisfaction Questionnaire (PSQ-9) was derived from the MA-PSQ18 to measure patients’ satisfaction with community pharmacists and pharmacies, following an exploratory factor analysis (EFA) conducted on a subsample (Sample 1) to refine and shorten the instrument (Sacre et al., 2023). Items were retained based on a stringent loading threshold of ≥ 0.700 to ensure strong construct representation while minimising respondent burden and improving feasibility in routine patient surveys (Boateng et al., 2018). The initial EFA revealed a two-factor structure explaining 64.0% of the total variance (Factor 1: 56.7%; Factor 2: 7.3%). Eight items loaded on Factor 1, of which six met the ≥ 0.740 criterion, and ten items loaded on Factor 2, of which three exceeded 0.821 (Supplemental Appendix A). The retained items captured key dimensions of satisfaction, including pharmacist communication, trust, time spent in care, affordability, and confidence in care accuracy. The preliminary selection was reviewed by an external committee of five pharmacists (two board-certified pharmacotherapy specialists, two community pharmacists, and one pharmacoepidemiologist), who independently evaluated the content validity, representativeness, and clarity of the items. Consensus agreement among committee members exceeded 95%. Based on their feedback, nine items were finalised to form the PSQ-9. A second EFA was subsequently conducted to re-examine and confirm the factor structure of the finalised scale.

### Ethical considerations

Ethical approval was granted by the Ethics and Research Committee of the School of Pharmacy, Lebanese International University (Protocol No. 2025ERC-009-LIUSOP). All participants provided informed consent before participation. The study followed the principles of the Declaration of Helsinki, ensuring confidentiality and ethical conduct throughout the research process.

### Sample size calculation

The required sample size was estimated using G*Power version 3.1.9.7 (Heinrich Heine University, Düsseldorf, Germany). For the planned multiple linear regression predicting PSQ-9 scores, a small effect size (f² = 0.0526; R² = 0.05), α = 0.05, power = 0.80, and 25 predictors indicated a minimum of 454 participants. For the validation analysis, a 10:1 participant-to-item ratio (Boateng et al., 2018) required 90 respondents for the nine PSQ-9 items, doubled to 180 for two independent subsamples. Accordingly, a minimum of 454 participants was deemed sufficient to meet both regression and validation requirements, ensuring 80% power and a 95% confidence level.

### Statistical analysis

All analyses were conducted in R version 4.5.1 (R Foundation for Statistical Computing, Vienna, Austria) using RStudio version 2025.09.1 + 401 (Cucumberleaf Sunflower, Posit Software, PBC). Descriptive statistics summarised participant sociodemographic, socioeconomic, and clinical characteristics, as well as patient-reported experiences. Continuous variables were expressed as means ± standard deviations (SD), and categorical variables as frequencies and percentages.

The total sample was randomly divided into two equal subsets. An initial EFA was performed on the first subset to guide PSQ-9 item selection and reduction, as detailed in the development section. A second EFA was then conducted within the same exploratory subsample (Sample 1) to re-examine and confirm the underlying structure of the retained items after item refinement. All EFAs were performed using the *psych* and *GPArotation* packages with Promax rotation, following verification of sampling adequacy via the Kaiser-Meyer-Olkin (KMO) measure and Bartlett’s test of sphericity. Factors with eigenvalues greater than 1 were retained. Subsequently, a confirmatory factor analysis (CFA) was conducted exclusively on the second independent subset (Sample 2) using the *lavaan* package with maximum likelihood estimation to validate the factor structure identified in the EFA. This sequential exploratory-confirmatory approach using independent subsamples is recommended in scale development to minimise overfitting and strengthen structural validity (Boateng et al., 2018). Model fit was assessed using χ²/df, Comparative Fit Index (CFI), Tucker-Lewis Index (TLI), Root Mean Square Error of Approximation (RMSEA), and Standardized Root Mean Square Residual (SRMR), considering acceptable fit at χ²/df < 3, CFI and TLI ≥ 0.95, RMSEA ≤ 0.08, and SRMR ≤ 0.08 (Hu & Bentler, 1999). The model was visualised and standardised loadings obtained with IBM SPSS Amos version 24. All remaining analyses were performed on the total sample. Measurement invariance of the PSQ-9 was tested using multigroup CFA across gender, health status, and access to healthcare. Configural, metric, and scalar invariance were examined, with invariance confirmed when ΔCFI ≤ 0.010, ΔRMSEA ≤ 0.015, and ΔSRMR ≤ 0.010 (Chen, 2007).

Reliability was evaluated through Pearson correlations among the total, subscale, and item scores, as well as Cronbach’s α (via *psych*) and McDonald’s ω (via *semTools*). Concurrent and discriminant validity were examined through correlations with the PEI, BCI, PPI, EQ-VAS, and LMAS-14 scores.

Associations between PSQ-9 scores and sociodemographic, socioeconomic, clinical, and patient-reported experience variables were first examined using univariable linear regression. Variables with *P* < 0.20 were then included in multivariable analyses using the backward AIC selection method (*MASS* package). Model 1 incorporated sociodemographic and socioeconomic predictors, Model 2 included clinical and experience-related factors, and Model 3 combined significant predictors from the first two models. Unstandardised (B) and standardised (β) coefficients, 95% confidence intervals (CIs), and *P* values were reported, with statistical significance set at *P* < 0.05.

## Results

### Sociodemographic and socioeconomic characteristics

The study total sample comprised 501 individuals with an average age of 34.7 ± 15.2 years. Females represented about two-thirds of participants (67.3%). Most respondents lived in South Lebanon (68.5%) or Beirut (23%). Over half were single (52.3%), and the majority held a university degree (81.6%). Employment was reported by 56.5%, whereas 40.3% were unemployed. Around one-third had a household income exceeding 1500 USD, while about 22% earned below 500 USD. The mean IFDFW score reached 47.9 ± 19.9. Detailed participant characteristics are summarised in Table 1.

**Table 1.** Sociodemographic and socioeconomic characteristics of the study participants.

Variable	Mean or Frequency	SD or %
*Age*	34.66	15.17
*Gender*		
Male	164	32.73
Female	337	67.27
*Region*		
Beirut	115	22.95
Bekaa	10	2.00
Mount Lebanon	15	2.99
North	18	3.59
South	343	68.46
*Marital status*		
Single	262	52.30
Married	216	43.11
Divorced/ Widowed	23	4.59
*Education level*		
Not educated	12	2.40
School level	80	15.97
University level	409	81.64
*Occupation*		
Unemployed	202	40.32
Employed/ Self-employed	283	56.49
Retired	16	3.19
*Monthly income*		
Less than 500 USD	110	21.96
501 - 999 USD	112	22.36
1000 - 1500 USD	119	23.75
More than 1500 USD	160	31.94
*Total IFDFW score*	47.94	19.92

SD: standard deviation; USD: US Dollars; IFDFW: InCharge Financial Distress/Financial Well-Being Scale.

### Clinical characteristics and patient-reported experiences with community pharmacy

Table 2 presents participants’ clinical profiles and reported experiences with community pharmacies. About 26% had at least one chronic condition, with averages of 1.33 ± 1.56 comorbidities and 1.04 ± 1.66 daily medications. Most participants (79.8%) indicated easy access to healthcare, and 61.7% had health coverage, mainly through private insurance or the National Social Security Fund. Regular pharmacist counselling was reported by 22.9%, with nearly half (47.1%) noting sessions of 5–10 min. Mean scores were 20.09 ± 2.01 for the PEI, 9.69 ± 2.70 for BCI, and 35.22 ± 6.51 for the PPI. The LMAS-14 mean was 36.66 ± 11.04, and the average EQ VAS score reached 76.16 ± 20.82.

**Table 2.** Clinical characteristics and patient-reported experiences with community pharmacies.

Variable	Mean or Frequency	SD or %
*Current health status*		
No chronic illness	371	74.05
Chronic illness	130	25.95
*Total number of comorbidities*	1.33	1.56
Number of chronic daily medications	1.04	1.66
*Easy access to healthcare*		
No	101	20.16
Yes	400	79.84
*Health coverage*		
No coverage	192	38.32
National Social Security Fund	84	16.77
Public insurance	57	11.38
Private insurance	168	33.53
*Do you receive regular counseling by a pharmacist?*		
No, not at all	128	25.55
Yes, from time to time	258	51.50
Yes, regularly	115	22.95
*Duration of counseling by a pharmacist, once received*		
Less than 5 min	221	44.11
5–10 min	236	47.11
More than 10 min	44	8.78
*Patient Expectation Index score*	20.09	2.01
*Barriers for Communication with Pharmacist score*	9.69	2.70
*Patient Perception Index score*	35.22	6.51
*LMAS-14 score*	36.66	11.04
*EQ VAS score*	76.16	20.82

SD: standard deviation; LMAS-14: 14-item Lebanese Medication Adherence Scale; EQ VAS stands for EuroQol Visual Analogue Scale.

### Validation of the PSQ-9

#### Exploratory factor analysis

Following item refinement, a second EFA was performed on Sample 1 (N = 250) to identify the latent structure of the final PSQ-9. All nine items were retained and analyzed using Promax rotation. No items demonstrated high intercorrelation (r > 0.9), weak loadings (< 0.30), or low communalities (< 0.30). Sampling adequacy was strong (KMO = 0.909), and Bartlett’s test confirmed factorability (*P* < 0.001).

The analysis revealed a two-factor structure with Eigenvalues above 1, explaining 71.33% of the variance. Factor 1 encompassed six items representing interpersonal care and professional engagement, while Factor 2 comprised three items reflecting affordability and informational assurance. The Promax-rotated component matrix is shown in Table 3.

**Table 3.** Promax rotated component matrix of the PSQ-9 (Sample 1).

PSQ-9 item #	MA-PSQ-18 item #	PSQ-9 items	Factor 1	Factor 2	h^2^
1	11	My pharmacists take care of me in a very friendly and courteous manner	0.924		0.759
2	13	Pharmacists do not ignore what I tell them	0.906		0.751
3	12	Pharmacists who provide my medical care are not too much in a hurry when they provide care for me	0.863		0.735
4	14	I trust the ability of the pharmacists who provide care for me	0.825		0.724
5	15	Pharmacists usually spend enough time with me	0.783		0.729
6	17	I am satisfied with the medical care I receive by pharmacists	0.754		0.692
7	7	I do not have to pay more than I can afford for my medical care in a community pharmacy		0.897	0.700
8	4	Pharmacists do not make me wonder if their provided medical care is correct		0.781	0.698
9	1	Pharmacists are good about explaining the reason for medical tests		0.757	0.631
		*Percentage of variance explained*	58.63%	12.70%	

Factor 1 = Interpersonal Care and Professional Engagement; Factor 2 = Affordability and Informational Assurance.

h^2^ = communalities.

Total percentage of variance explained: 71.33%.

Kaiser-Meyer-Olkin (KMO) = 0.909.

Bartlett's test of sphericity: *P* < 0.001.

#### Confirmatory factor analysis

**Figure 1.**
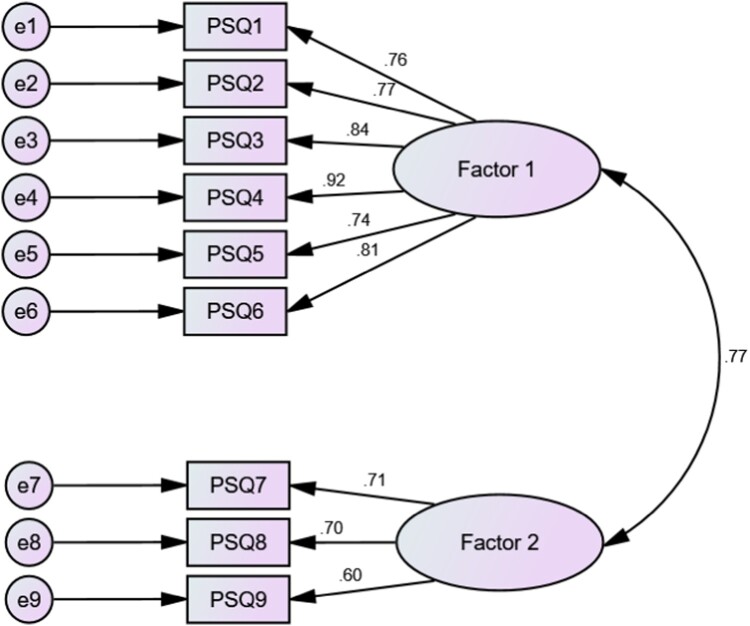
Standardised estimates of factor loadings from the confirmatory factor analysis (CFA) of the PSQ-9 items in sample 2. Factor 1 = Interpersonal Care and Professional Engagement; Factor 2 = Affordability and Informational Assurance. e = error term.

A CFA was performed on Sample 2 (N = 251) to confirm the two-factor structure of the PSQ-9 derived from the EFA. The model demonstrated good fit: χ²/df = 46.260/26 = 1.779 (*P* = 0.009). Fit indices further supported the model adequacy, with CFI = 0.984 and TLI = 0.978, both above 0.95. The RMSEA was 0.056 (90% CI: 0.028–0.082; *P* = 0.331), and the SRMR was 0.027, indicating an excellent overall fit. Figure 1 displays the standardised factor loadings and structural paths of the PSQ-9 model.

#### Measurement invariance

Table 4 presents the multigroup CFA measurement invariance results of the PSQ-9 in the total sample across gender, health status, and access to healthcare. For all three grouping variables, the configural, metric, and scalar models demonstrated acceptable fit, with minimal changes in fit indices (ΔCFI ≤ 0.010; ΔRMSEA ≤ 0.015; ΔSRMR ≤ 0.010).

**Table 4.** Measurement invariance of the PSQ-9 across gender, health status, and access to healthcare groups.

Model	CFI	RMSEA	SRMR	Model comparison	ΔCFI	ΔRMSEA	ΔSRMR
**Model 1: across gender (male vs. female)**							
Configural	0.999	0.054	0.031				
Metric	0.998	0.055	0.040	Configural vs metric	0.001	0.001	0.009
Scalar	0.999	0.051	0.032	Metric vs scalar	0.001	0.004	0.008
**Model 2: across current health status (chronic illness vs. no illness)**							
Configural	0.984	0.057	0.039				
Metric	0.984	0.054	0.042	Configural vs metric	< 0.001	0.003	0.003
Scalar	0.984	0.049	0.042	Metric vs scalar	< 0.001	0.005	< 0.001
**Model 3: across easy access to healthcare (yes vs. no)**							
Configural	0.982	0.059	0.031				
Metric	0.981	0.058	0.039	Configural vs metric	0.001	0.001	0.008
Scalar	0.979	0.057	0.041	Metric vs scalar	0.002	0.001	0.002

CFI: Comparative Fit Index; RMSEA: Root Mean Square Error of Approximation; SRMR: Standardized Root Mean Square Residual.

#### Reliability and internal consistency

**Figure 2.**
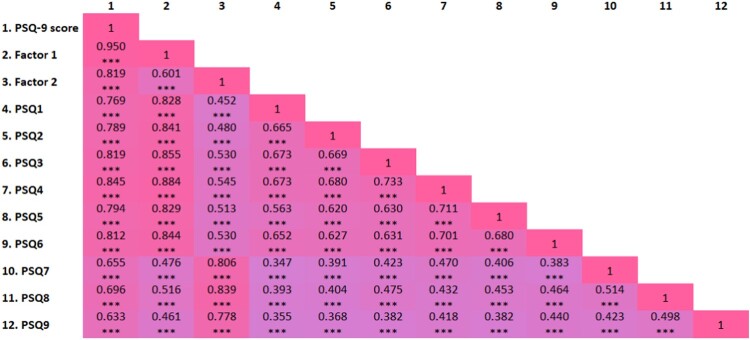
Pearson correlation matrix for the PSQ-9 items, factor subscales, and total score. PSQ-9: 9-item Patient Satisfaction Questionnaire; Factor 1 (Interpersonal Care and Professional Engagement); Factor 2 (Affordability and Informational Assurance); PSQ1 to PSQ9 (PSQ-9 scale item numbers). ***P < 0.001.

Figure 2 presents Pearson correlations among the PSQ-9 total, its two subscales, and items (full sample). All coefficients were significant (*P* < 0.001), indicating strong internal consistency. The subscales correlated highly with the total (Factor 1: r = 0.950; Factor 2: r = 0.819). Item-subscale correlations were high (Factor 1 items: r = 0.828–0.884; Factor 2 items: r = 0.778–0.839), confirming factor coherence.

Table 5 summarises the reliability outcomes for the PSQ-9 and its two factors. Cronbach’s α for the PSQ-9 was 0.904, with subscales ranging from 0.733 to 0.921. McDonald’s ω reached 0.910 for the overall scale, 0.921 for Factor 1, and 0.736 for Factor 2. Test-retest reliability showed good stability, with ICC = 0.729 (95% CI: 0.676–0.772; *P* < 0.001) for the total score, and ICCs ranging from 0.685 to 0.725 for the two subscales, all *P* < 0.001.

**Table 5.** Reliability indices of the PSQ-9 total scale and its subscales, encompassing measures of internal consistency and test-retest stability.

Scale / Subscale	Internal consistency reliability		Test-retest reliability			
	Cronbach’s α	McDonald’s ω	ICC	Lower 95% CI	Upper 95% CI	*P* value
PSQ-9	0.904	0.910	0.729	0.676	0.772	< 0.001
Factor 1	0.921	0.921	0.685	0.625	0.736	< 0.001
Factor 2	0.733	0.736	0.725	0.672	0.769	< 0.001

PSQ-9 = 9-item Patient Satisfaction Questionnaire; Factor 1 = Interpersonal Care and Professional Engagement; Factor 2 = Affordability and Informational Assurance; ICC = intraclass correlation coefficient; CI = confidence interval.

#### Concurrent and discriminant validity

Concurrent validity was demonstrated through significant associations between the PSQ-9 score and the PEI (r = 0.164, *P* < 0.001), BCI (r = –0.160, *P* < 0.001), and PPI (r = 0.226, *P* < 0.001) scores. In contrast, discriminant validity was indicated by the absence of significant relationships between the PSQ-9 and both the EQ-VAS (r = 0.019, *P* = 0.679) and LMAS-14 scores (r = 0.052, *P* = 0.243).

### Assessment of patient satisfaction and its associated factors

The mean satisfaction score on the PSQ-9 was 35.00 ± 5.30. Univariable linear regression analyses indicated that several variables were significantly associated with patient satisfaction. Higher satisfaction was observed among older participants (B = 0.032, *P* = 0.038), those residing in the Bekaa region (B = 4.200, *P* = 0.016), married individuals (B = 1.097, *P* = 0.024), and retired participants (B = 3.787, *P* = 0.006). Satisfaction also increased with higher IFDFW scores (B = 0.039, *P* = 0.001), easier access to healthcare (B = 2.359, *P* < 0.001), private health insurance (B = 1.670, *P* = 0.003) receiving counselling from time to time (B = 2.330, *P* < 0.001) or regularly (B = 3.699, *P* < 0.001), and longer counselling durations (>10 min; B = 3.796, *P* < 0.001). Additionally, higher PEI (B = 0.432, *P* < 0.001) and PPI (B = 0.184, *P* < 0.001) scores were positively associated with satisfaction. Conversely, participants from the South region (B = –1.223, *P* = 0.031), those with university education (B = –3.468, *P* = 0.026), and those reporting greater barriers to communication (BCI) (B = –0.313, *P* < 0.001) had significantly lower satisfaction levels. The detailed results of the univariable linear regression analyses are summarised in Table 6.

**Table 6.** Univariable linear regression of sociodemographic, socioeconomic, clinical, and patient-reported experience variables associated with the PSQ-9 score.

Variable	B	β	95% CI		*P* value
			Lower	Upper	
*Age*	0.032	0.093	0.002	0.063	0.038
*Gender*					
Female vs. Male	0.193	0.036	−0.800	1.186	0.702
*Region* (reference: Beirut)					
Bekaa	4.200	0.792	0.802	7.598	0.016
Mount Lebanon	−1.667	−0.314	−4.496	1.163	0.248
North	0.144	0.027	−2.468	2.757	0.914
South	−1.223	−0.231	−2.333	−0.112	0.031
*Marital Status* (reference: Single)					
Married	1.097	0.207	0.143	2.052	0.024
Divorced/ Widowed	0.129	0.024	−2.130	2.388	0.911
*Education Level* (reference: Not educated)					
School level	−3.133	−0.591	−6.349	0.082	0.056
University level	−3.468	−0.654	−6.510	−0.425	0.026
*Occupation* (reference: Unemployed)					
Employed/ Self-employed	−0.035	−0.007	−0.989	0.919	0.942
Retired	3.787	0.714	1.096	6.477	0.006
*Monthly income* (reference: Less than 500 USD)					
501 - 999 USD	−0.776	−0.146	−2.173	0.621	0.276
1000 - 1500 USD	0.341	0.064	−1.036	1.718	0.627
More than 1500 USD	0.495	0.093	−0.794	1.784	0.451
*IFDFW score*	0.039	0.147	0.016	0.062	0.001
*Current health status*					
Chronic illness vs. No illness	1.046	0.197	−0.013	2.106	0.053
*Total number of comorbidities*	0.154	0.045	−0.145	0.452	0.312
*Number of chronic daily medications*	0.267	0.084	−0.013	0.547	0.062
*LMAS-14 score*	0.025	0.052	−0.017	0.067	0.243
*Easy access to healthcare*					
Yes vs. No	2.359	0.445	1.216	3.502	< 0.001
*Health coverage* (reference: No coverage)					
National Social Security Fund (NSSF)	1.265	0.238	−0.089	2.619	0.067
Public insurance	0.374	0.070	−1.187	1.935	0.638
Private insurance	1.670	0.315	0.576	2.763	0.003
*Do you receive regular counseling by a pharmacist?* (reference: No, not at all)					
Yes, from time to time	2.330	0.439	1.236	3.423	< 0.001
Yes, regularly	3.699	0.697	2.4	4.998	< 0.001
*Duration of counseling by a pharmacist, once received* (reference: Less than 5 min)					
5–10 min	0.939	0.177	−0.019	1.897	0.055
More than 10 min	3.796	0.716	2.106	5.487	< 0.001
*Patient Expectation Index score*	0.432	0.164	0.204	0.661	< 0.001
*Barriers for Communication with Pharmacist score*	−0.313	−0.16	−0.484	−0.143	< 0.001
*Patient Perception Index score*	0.184	0.226	0.114	0.253	< 0.001
*EQ VAS score*	0.005	0.019	−0.018	0.027	0.679

B: Unstandardized Beta; β: Standardized Beta; 95% CI: 95% confidence interval; USD: US Dollars; IFDFW: InCharge Financial Distress/Financial Well-Being Scale.

#### Multivariable analysis

Table 7 presents three multivariable linear regression models examining predictors of patient satisfaction. In Model 1, which included sociodemographic and socioeconomic variables, higher satisfaction was associated with residence in the Bekaa region (B = 4.776, *P* = 0.009), being retired (B = 3.420, *P* = 0.012), and having a higher IFDFW score (B = 0.040, 0.001). In Model 2, which included clinical characteristics and patient-reported experiences, satisfaction was greater among participants with easier access to healthcare (B = 1.675, P 0.003), those receiving counselling from time to time (B = 1.636, *P* = 0.003) or regularly (B = 1.961, *P* = 0.005), and those reporting longer counselling time (>10 min; B = 2.643, *P* = 0.002), higher PEI (B = 0.352, *P* = 0.002), and higher PPI (B = 0.128, *P* < 0.001). Conversely, greater barriers to communication (BCI) were associated with lower satisfaction (B = –0.244, *P* = 0.004). In Model 3, which combined significant predictors from the previous models, satisfaction remained positively associated with residence in the Bekaa region (B = 4.303, *P* = 0.009), easier access to healthcare (B = 1.470, *P* = 0.012), receiving counselling from time to time (B = 1.616, *P* = 0.004) or regularly (B = 1.864, *P* = 0.008), longer counselling time (>10 min; B = 2.441, *P* = 0.004), higher PEI (B = 0.356, *P* = 0.001), and higher PPI (B = 0.110, *P* = 0.002), while greater barriers to communication continued to predict lower satisfaction (B = –0.235, *P* = 0.005).

**Table 7.** Multivariable linear regression of factors predicting patient satisfaction, with the PSQ-9 score serving as the dependent variable.

Variable	B	β	95% CI		*P* value
			Lower	Upper	
Model 1: including sociodemographic and socioeconomic characteristics*					
*Region* (reference: Beirut)					
Bekaa	4.776	0.901	1.377	8.176	0.006
Mount Lebanon	−1.111	−0.209	−3.961	1.739	0.444
North	−0.045	−0.008	−2.637	2.547	0.973
South	−0.924	−0.174	−2.050	0.202	0.108
*Occupation* (reference: Unemployed)					
Employed/ Self-employed	−0.051	−0.010	−1.008	0.906	0.917
Retired	3.420	0.645	0.744	6.095	0.012
*IFDFW score*	0.040	0.149	0.016	0.063	0.001
Model 2: including clinical characteristics and patient-reported experiences**					
*Easy access to healthcare*					
Yes vs. No	1.675	0.316	0.577	2.774	0.003
*Do you receive regular counseling by a pharmacist?* (reference: No, not at all)					
Yes, from time to time	1.636	0.308	0.547	2.726	0.003
Yes, regularly	1.961	0.369	0.590	3.332	0.005
*Duration of counseling by a pharmacist, once received* (reference: Less than 5 min)					
5–10 min	0.248	0.047	−0.690	1.186	0.604
More than 10 min	2.643	0.498	0.990	4.296	0.002
*Patient Expectation Index score*	0.352	0.133	0.135	0.568	0.002
*Barriers for Communication with Pharmacist score*	−0.244	−0.124	−0.409	−0.078	0.004
*Patient Perception Index score*	0.128	0.158	0.060	0.196	< 0.001
Model 3: including statistically significant sociodemographic, socioeconomic, clinical, and patient-reported experience characteristics from Model 1 and Model 2***					
*Region* (reference: Beirut)					
Bekaa	4.303	0.811	1.086	7.519	0.009
Mount Lebanon	−0.818	−0.154	−3.551	1.915	0.557
North	0.444	0.084	−1.996	2.884	0.721
South	−0.492	−0.093	−1.567	0.583	0.369
*IFDFW score*	0.021	0.077	−0.003	0.044	0.087
*Easy access to healthcare*					
Yes vs. No	1.470	0.277	0.330	2.611	0.012
*Do you receive regular counseling by a pharmacist?* (reference: No, not at all)					
Yes, from time to time	1.616	0.305	0.529	2.703	0.004
Yes, regularly	1.864	0.351	0.492	3.236	0.008
*Duration of counseling by a pharmacist, once received* (reference: Less than 5 min)					
5–10 min	0.251	0.047	−0.685	1.188	0.598
More than 10 min	2.441	0.460	0.781	4.102	0.004
*Patient Expectation Index score*	0.356	0.135	0.140	0.572	0.001
*Barriers for Communication with Pharmacist score*	−0.235	−0.120	−0.400	−0.07	0.005
*Patient Perception Index score*	0.110	0.135	0.041	0.179	0.002

B: Unstandardized Beta; β: Standardized Beta; 95% CI: 95% confidence interval; IFDFW: InCharge Financial Distress/Financial Well-Being Scale.

*Variables initially entered into the model: age; region; marital status; education Level; occupation; IFDFW score.

**Variables initially entered into the model: current health status; number of chronic daily medications; easy access to healthcare; health coverage; do you receive regular counselling by a pharmacist?; duration of counselling by a pharmacist, once received; Patient Expectation Index score; Barriers for Communication with Pharmacist score; Patient Perception Index score.

***Variables initially entered into the model: region; occupation; IFDFW score; easy access to healthcare; do you receive regular counselling by a pharmacist?; duration of counselling by a pharmacist, once received; Patient Expectation Index score; Barriers for Communication with Pharmacist score; Patient Perception Index score.

## Discussion

This study developed and validated the PSQ-9, a concise instrument to measure patient satisfaction in community pharmacy settings. The results demonstrate strong psychometric properties and confirm that the PSQ-9 captures the main dimensions of the pharmacy experience in a brief and practical format. The two identified factors, Interpersonal and Professional Engagement, and Affordability and Informational Assurance align with domains of established satisfaction scales such as the PSPSQ 2.0, the MacKeigan-Larson scale, and the Pharmaceutical Care Satisfaction Questionnaire, all of which emphasise communication, accessibility, and trust as core components of satisfaction (Larson et al., 2002; MacKeigan & Larson, 1989; Sakharkar et al., 2015). The PSQ-9 therefore preserves the conceptual framework of prior instruments while combining core domains to offer a time-efficient and contextually relevant tool.

Psychometrically, the PSQ-9 showed excellent internal consistency, temporal stability, and model fit. The two-factor solution was confirmed in the CFA and accounted for a high proportion of variance. Measurement invariance was demonstrated across gender, health status, and healthcare access, a notable strength and, to our knowledge, not previously reported in patient satisfaction scales. This indicates that the PSQ-9 measures satisfaction equivalently across subgroups, allowing fair and meaningful comparisons, an essential property for quality-assessment tools in diverse populations.

Concurrent and discriminant patterns reinforce construct coherence. The PSQ-9’s positive associations with PEI and PPI, and its negative association with communication barriers, follow the expectancy–disconfirmation framework observed in outpatient studies, where communication quality and interpersonal warmth are key determinants of satisfaction (Kamei et al., 2001). The weak or absent associations with global health status and medication adherence confirm that satisfaction reflects perceptions of service experience in a community pharmacy rather than clinical outcomes, consistent with evidence that satisfaction primarily depends on relational and system features rather than health status itself (Bleich et al., 2009; Sofaer & Firminger, 2005).

When compared with existing tools, the PSQ-9 shares relational and informational domains found in longer scales but uniquely integrates affordability, often under-represented in Western instruments. Similar to the PSPSQ 2.0, the first factor captures professional behaviour and pharmacist engagement, while the second extends beyond technical quality to include cost and clarity of information, key determinants in low- and middle-income settings where medication affordability and transparency strongly influence satisfaction (Albabtain et al., 2024). Shorter international adaptations, such as the Chinese 6-item and Arabic PSPSQ versions, have similarly emphasised these practical and communicative elements (Albabtain et al., 2024; Chien et al., 2025). Thus, the PSQ-9 maintains conceptual equivalence while embedding the socioeconomic context of Lebanon and comparable health systems.

In the multivariable analysis, Model 1 identified region and financial well-being as significant predictors. Participants from the Bekaa reported higher satisfaction, possibly reflecting closer pharmacist-patient relationships or lower baseline expectations in less urban areas. Comparable regional trends have been reported in Greece and the United States (Karakolias et al., 2024; Malewski et al., 2015). Higher financial well-being was also associated with greater satisfaction, echoing the IMPHACT-LB Quality-of-Life study, where financial strain negatively influenced perceived care and well-being (Hajj et al., 2025). These findings confirm that economic stability and social context shape how community pharmacy services are evaluated.

Model 2, which included both experiential and perceptual variables, showed that ease of access, pharmacist counselling (frequency and duration), higher expectations, and better perceived pharmacist performance were associated with higher satisfaction, while communication barriers predicted lower satisfaction. These results are consistent with studies from Saudi Arabia and Jordan reporting that accessibility, waiting time, and pharmacist interaction strongly affect satisfaction (Al-Tannir et al., 2016; Naser & Abu Sbeat, 2022). The influence of counselling quality and duration agrees with research from other contexts (Ali et al., 2022; Kucukarslan & Nadkarni, 2008). The persistence of both experiential and perceptual effects suggests that satisfaction results from patients’ lived experiences as well as their interpretations of those experiences.

Model 3, which combined significant predictors from Models 1 and 2, confirmed the robustness of these associations. Satisfaction remained higher among participants from Bekaa, those with better financial well-being, easier access to healthcare, and more frequent or longer pharmacist counselling. Similarly, higher expectations and positive perceptions continued to enhance satisfaction, whereas communication barriers reduced it. This integrated model illustrates how satisfaction emerges from the dynamic interplay of contextual, process, and perceptual dimensions, aligning with Donabedian’s structure-process-outcome framework (Donabedian, 1988). Together, these findings portray a layered understanding of satisfaction. Contextual factors set expectations; experiential factors define daily interactions; and perceptual factors translate these experiences into judgments. The PSQ-9’s two factors correspond closely to these layers, providing a clear framework for targeted community pharmacy service improvement.

### Implications for practice and policy

The PSQ-9 offers a validated, easy-to-use instrument for assessing community-pharmacy performance and patient experience. Its brevity supports its use in surveys or digital feedback forms. Pharmacies can apply it regularly to identify strengths such as pharmacist professionalism and gaps such as counselling time or communication clarity. Policymakers can use PSQ-9 results to benchmark satisfaction across regions and socioeconomic groups. Future studies should examine its responsiveness to quality-improvement interventions and its predictive value for adherence or loyalty. Validation in other populations would further enhance its utility.

### Strengths and limitations

This study has several strengths. It used a robust, multi-stage validation design with independent EFA and CFA samples. The large and geographically diverse sample enhances generalizability. Demonstrating measurement invariance across gender, health status, and access to care is a novel and valuable contribution. The inclusion of socioeconomic, experiential, and perceptual predictors in sequential models provided a comprehensive view of determinants.

However, limitations must be noted. The cross-sectional design precludes causal inference. Online data collection may have overrepresented younger and more educated respondents. Satisfaction was self-reported and potentially influenced by social desirability bias. Regional sample sizes were unequal, which may affect precision of estimates. Future work should adopt stratified sampling and longitudinal designs to confirm temporal stability and responsiveness.

## Conclusion

The PSQ-9 is a valid, reliable, and culturally adapted measure capturing essential domains of patient satisfaction with community pharmacy services. Its two-factor structure parallels established satisfaction constructs while reflecting social and economic context. The demonstration of measurement invariance strengthens its use for subgroup and regional comparisons. Counselling quality, accessibility, and financial well-being emerged as key correlates, showing that both interpersonal interaction and contextual realities shape satisfaction. The PSQ-9 can thus serve as a practical, standardised tool to support patient-centered and equitable pharmacy care in the community setting.

## Supplementary Material

Supplemental Material Appendix

## Data Availability

The dataset associated with this article is available from the corresponding author upon reasonable request.
