# Supplementary material for: Development and validation of the 9-item Patient Satisfaction Questionnaire (PSQ-9) for use in community pharmacy settings
Source: J Pharm Policy Pract. 2026 Jun 17;19(1):2686185. doi: 10.1080/20523211.2026.2686185 (PMC13276807; doi:10.1080/20523211.2026.2686185)
Supplement: Supplemental Material Appendix [file JPPP_A_2686185_SM1004.pdf]

Exploratory Factor Analysis for Item Reduction and Selection of the MA-PSQ18

MA-PSQ-18 Items	Factor 1	Factor 2
11. My pharmacists take care of me in a very friendly and courteous manner	0.977	
13. Pharmacists do not ignore what I tell them	0.965	
12. Pharmacists who provide my medical care are not too much in a hurry when they provide care for me	0.886	
14. I trust the ability of the pharmacists who provide care for me	0.863	
15. Pharmacists usually spend enough time with me	0.797	
17. I am satisfied with the medical care I receive by pharmacists	0.740	
18. I am able to get medical care by my pharmacists whenever I need it	0.542	
8. I have easy access to competent pharmacists as I need	0.485	
7. I do not have to pay more than I can afford for my medical care in a community pharmacy		0.991
4. Pharmacists do not make me wonder if their provided medical care is correct		0.842
1. Pharmacists are good about explaining the reason for medical tests		0.821
3. The medical care I have been receiving by my pharmacist is just about perfect		0.644
9. Where I get pharmaceutical care, people do not have to wait too long for emergency treatment		0.609
5. I feel confident that I can get the medical care I need from my pharmacy without being set back financially		0.607
6. When I go to the community pharmacy, they are careful to check everything when providing medical care for me		0.527
10. Pharmacists are not too businesslike and impersonal toward me		0.506
16. It's easy to get access for medical care right away in a community pharmacy	0.412	0.436
2. I think my community pharmacy has everything needed to provide complete medical care		0.426
% of variance explained	56.74%	7.28%
Total % of variance explained	64.02%	
Kaiser-Meyer-Olkin Measure of Sampling Adequacy	0.952	
Bartlett's Test of Sphericity	P < 0.001	
